# Effects of temperature on mating behaviour and mating success: A meta‐analysis

**DOI:** 10.1111/1365-2656.13761

**Published:** 2022-07-10

**Authors:** Natalie Pilakouta, Anaїs Baillet

**Affiliations:** ^1^ School of Biological Sciences University of Aberdeen Aberdeen UK; ^2^ Observatoire des Sciences de l'Univers de Rennes (OSUR) Université de Rennes Rennes France; ^3^ Department of Wood and Forest Sciences Laval University Quebec QC Canada

**Keywords:** choosiness, climate change, mate choice, mating latency, meta‐analysis, sexual selection, thermal effects

## Abstract

1. In light of global climate change, there is a pressing need to understand how populations will respond to rising temperatures. Understanding the effects of temperature changes on mating behaviour is particularly important, given its implications for population viability.
2. To this end, we performed a meta‐analysis of 53 studies to examine how temperature changes influence mating latency, choosiness and mating success. We hypothesized that if higher temperatures make mate searching and mate assessment more costly due to an elevated metabolism, this may lead to a reduction in mating latency and choosiness, thereby increasing overall mating success.
3. We found no evidence for an overall effect of temperature on mating latency, choosiness, or mating success. There was an increase in mating success when animals were exposed to higher temperatures during mating trials but not when they were exposed before mating trials.
4. In addition, in a subset of studies that measured both mating latency and mating success, there was a strong negative relationship between the effect sizes for these traits. This suggests that a decrease in mating latency at higher temperatures was associated with an increase in mating success and vice versa.
5. In sum, our meta‐analysis provides new insights into the effects of temperature on mating patterns. The absence of a consistent directional effect of temperature on mating behaviours and mating success suggests it may be difficult to predict changes in the strength of sexual selection in natural populations in a warming world. Nevertheless, there is some evidence that (a) higher temperatures during mating may lead to an increase in mating success and that (b) an increase in mating success is associated with a decrease in mating latency.

## Graphical abstract

In a meta‐analysis examining how temperature changes influence mating behaviour and mating success the authors found: (a) higher temperatures during mating may lead to an increase in mating success and that (b) an increase in mating success is associated with a decrease in mating latency.

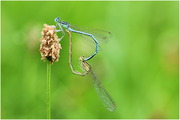

## Abstract (fr)

1. En prévision du changement climatique, il est nécessaire de comprendre comment les populations répondent à l'augmentation de la température. Appréhender les effets des variations de température sur le comportement d'accouplement devient important, étant donné le rôle de ce phénomène sur la viabilité des populations.
2. Cette méta‐analyse rassemble 53 études, dans le but d'évaluer comment les variations de températures influencent la latence pré‐accouplement, la sélectivité du partenaire sexuel et le succès d'accouplement. Selon notre hypothèse, l'augmentation de la température induirait un coût plus élevé de la recherche et de l'estimation du partenaire, dû à un métabolisme accéléré. Ceci pourrait conduire à une réduction de la latence d'accouplement et de la sélectivité, augmentant ainsi le succès d'accouplement.
3. L'analyse n'a montré aucun effet significatif de la température sur les trois traits étudiés. Le succès d'accouplement augmente lorsque les organismes sont exposés à des températures élevées, seulement lors des essais d'accouplement. Ce n'est pas le cas lorsqu'ils y sont exposés préalablement aux essais.
4. En considérant les études qui mesuraient à la fois la latence et le succès d'accouplement, l'analyse a montré une relation négative importante entre les effets moyens de ces deux traits. La diminution de la latence d'accouplement, à des températures élevées, serait alors associée à une augmentation du succès d'accouplement, et inversement.
5. La méta‐analyse offre de nouvelles perspectives quant aux effets de la température sur les tendances d'accouplement. L'absence d'un effet directionnel systématique de la température sur les comportements et succès d'accouplement suggère qu'en situation de changement climatique, les variations de la pression de la sélection sexuelle sur les populations naturelles sont difficiles à prédire. Cependant, les tendances montrent que (i) pendant l'accouplement, des températures plus élevées engendrent une augmentation du succès d'accouplement, et (ii) l'augmentation de ce dernier est associée à une diminution de la latence d'accouplement.

## 1 INTRODUCTION

In light of global climate change, there is a pressing need to understand how populations will respond and adapt to rising temperatures (Crozier & Hutchings, 2014). Because animal behaviour is particularly labile, there is a growing body of literature investigating the effects of temperature on a wide range of behavioural traits (Abram et al., 2017). Understanding how temperature changes might affect mating behaviour and mating success is particularly important given its link to population viability and performance (Candolin & Heuschele, 2008). Strong sexual selection can increase population fitness and reduce the risk of extinction (Moller & Alatalo, 1999, Lorch et al., 2003, Price et al., 2010, Lumley et al., 2015, Cally et al., 2019; but see Tanaka, 1996). Sexual selection could therefore play a major role in the capacity of populations to cope with climate change if stronger mate preferences for ‘good genes’ can lead to higher‐quality offspring (Candolin & Heuschele, 2008; Godwin et al., 2020; Martinossi‐Allibert et al., 2019). In order to better understand the link between temperature and the strength and direction of sexual selection, we need to focus on the different components of reproductive behaviour, such as choosiness and mating latency, to identify the specific underlying mechanisms influenced by temperature variation.

The effects of temperature on mating behaviour can be direct or indirect and are mediated through a wide range of physiological and sensory pathways (García‐Roa et al., 2020). For example, higher temperatures can increase the effectiveness of synaptic transmission and frequency of neuronal firing, resulting in faster decision‐making at a fine temporal scale in both ectotherms and endotherms (Fujii et al., 2002; Reig et al., 2010; Thompson et al., 1985; Volgushev et al., 2000). Temperature is also a key determinant of metabolic rate and locomotor performance, particularly in ectotherms (Gibert et al., 2001; Lachenicht et al., 2010). In turn, metabolic rate is closely linked to activity levels (Gunderson & Leal, 2015; Kearney et al., 2010) and can thus influence mate searching and spatio‐temporal distributions of the two sexes (García‐Roa et al., 2020). As a result, temperature has been shown to modulate a wide range of precopulatory mating behaviours, including mating latency, mate choice, courtship behaviour, remating rate and the intensity of intrasexual competition (e.g. Conrad et al., 2017; Gudka et al., 2019; Jiao et al., 2009; Katsuki & Miyatake, 2009; Kvanermo, 1998).

Here, we performed a meta‐analysis examining how an increase in temperature influences mating latency, choosiness and mating success. We focused on plastic, rather than evolutionary, responses to changes in temperature because there are far fewer studies on the latter. Two possible outcomes for our meta‐analysis were as follows: (a) if higher temperatures make mate searching and mate assessment more energetically costly due to an elevated metabolic rate, this might lead to a reduction in mating latency and cause females to be less choosy, thereby indirectly increasing overall mating success; (b) alternatively, if the benefits of mate choice are higher under warmer conditions (Leith et al., 2021; Qvarnström, 2001; Robinson et al., 2012), we might expect an increase in mating latency and choosiness, along with a decrease in mating success at the population level. In the former scenario, a reduction in mating latency and choosiness would lead to weaker sexual selection, whereas in the latter scenario an increase in mating latency and choosiness would lead to stronger sexual selection. Stronger sexual selection may in turn improve population viability by purging deleterious alleles in males that are also deleterious in females (McGuigan et al., 2011; Whitlock & Agrawal, 2009).

In addition, we examined whether the relationship between temperature and mating behaviour or mating success depends on the magnitude of the temperature change, the life stage during which the temperature change occurred and the duration of the temperature change. We expected stronger effects of temperature on mating behaviour and mating success when the change in temperature was larger and when it represented a long‐term change in thermal conditions. We might also expect stronger effects when the change in temperature occurs during mating trials, because it would directly affect metabolism, activity levels and locomotor performance during mate searching and assessment (García‐Roa et al., 2020). On the other hand, temperature changes in early development may have a more pronounced effect on mating behaviour than temperature changes in adulthood, since ‘critical windows’ in early life can affect development with long‐term consequences for behaviour (e.g. Adewale et al., 2011; Iossa et al., 2019; O'Connor et al., 2021).

The ability to generate general predictions for how populations will respond to climate change is crucial for management and conservation efforts, but it is difficult for individual empirical studies to address this issue, as they generally focus on a single species (Crozier & Hutchings, 2014). We suggest that meta‐analytical approaches present a powerful tool for better understanding and predicting the effects of rising temperatures on natural populations. By synthesizing data from published studies, meta‐analyses can detect common patterns across species (Harrison, 2011). Thus, in the context of predicting population responses to climate change, if a meta‐analysis reveals consistent directional effects of temperature on a particular trait across a wide range of taxonomic groups, it may allow us to generate some general predictions.

## 2 MATERIALS AND METHODS

### 2.1 Search protocol and data collection

This meta‐analysis was conducted following the preferred reporting items for systematic reviews and meta‐analysis (PRISMA) approach (Moher et al., 2009; Figure S1). Database searches were conducted using Web of Science and Scopus on the 20th and 21st of February 2020, respectively. We used search terms that would identify studies focusing on temperature variation and mating latency, choosiness, or mating success: (‘temperature’ OR ‘thermal’ OR ‘warm*’ OR ‘cold*’) AND (‘latency’ OR ‘mate’ or ‘mating’ OR ‘mate choice’ OR ‘choosy’ OR ‘choosiness’ OR ‘mat* preference’ OR ‘copulat*’ or ‘mating success’). All papers identified through these searches were checked for relevance based on the title and abstract (Figure S1). After removing papers that were clearly not relevant to our question, we screened the full text of the remaining papers to find studies that contained information on mating latency, choosiness, and/or mating success under two or more temperature conditions. We considered mating latency to be a measure of individuals' propensity to mate, which was described using different terms across papers (e.g. time to copulation, time to mating, premating period, precopulation period). We defined choosiness as ‘the change in mating propensity in response to alternative stimuli’ (Reinhold & Schielzeth, 2015). Choosiness thus referred to the strength of the mating preference when individuals were choosing between two potential mates (e.g. small vs. large) or sexual signals (e.g. mating calls). Mating success referred to the proportion of experimental pairs that engaged in copulation during the mating trial.

We included studies with either short‐term or long‐term exposure to different constant temperatures within a generation. Because our aim was to examine plastic, rather than evolutionary, responses to temperature, we excluded experimental evolution studies where organisms were exposed to contrasting thermal environments over multiple generations. We also excluded studies where there were confounding variables, for example when the temperature treatments were coupled with other factors, such as humidity. For studies on mating latency and choosiness, we only included those where the choosing sex (usually the female) was subjected to a temperature treatment (Table S1). For studies on mating success, we included studies where the male, female, or both were subjected to a temperature treatment (Table S1).

Our full‐text screening also included a small number of additional references that were not identified through our literature search and were instead obtained from other sources, such as a request for relevant papers from colleagues on Twitter (*n* = 10). Data from two of these papers were deemed relevant and included in the final analysis. Overall, full‐text screening identified 62 studies that met the experimental design criteria for inclusion in our meta‐analysis (Figure S1).

### 2.2 Effect size calculation

To calculate effect sizes, we extracted data from the main text, tables, or figures using the image analysis software WebPlotDigitzer (Rohatgi, 2019). We were unable to extract appropriate effect sizes from 13 studies due to missing test statistics or sample sizes. In these cases, we contacted the corresponding authors of these studies using a standardized email asking for the missing information. Seven of these authors responded to our email, and of those, four were able to provide the information needed to calculate effect sizes.

For each of the studies included in our meta‐analysis (*n* = 53), we calculated *r* effect sizes (correlation coefficient). In our analysis, a positive *r* effect size indicates that temperature and the trait of interest are positively correlated (e.g. higher temperatures are associated with increased mating success). For studies where temperature was treated as a continuous variable, we calculated *r* effect sizes directly from those data. For studies where temperature was treated as a categorical variable (e.g. ‘cold’ vs. ‘warm’ treatments), we first calculated Cohen's *d* and then converted that to an *r* effect size correlation coefficient. Effect sizes based on test statistics, such as chi‐square or *t*‐test, were calculated using the equations provided in Borenstein et al. (2011). Some studies included multiple effect sizes for the same or different species; to control for this, we included ‘study’ as a random factor in our analysis (see Section 2.3 for details). Overall, we collected 29 effect sizes for mating latency, 29 effect sizes for choosiness and 58 effect sizes for mating success (Table 1). These data comprised both vertebrates (amphibians, birds, fishes, reptiles) and invertebrates (arachnids, crustaceans, insects, molluscs) with insects being the most common taxonomic group studied (Table 1).

**TABLE 1:** Number of studies (*n*), species and effect sizes (*k*) used in our meta‐analysis on the effects of temperature on mating latency, choosiness (strength of preference) and mating success. We also show the breakdown by taxonomic group for each trait.

	Sample sizes		
	Studies (*n*)	Species	Effect sizes (*k*)
**Mating latency**	19	14	29
Arachnid	1	1	1
Insect	17	12	27
Mollusc	1	1	1
**Choosiness**	14	14	29
Amphibian	1	1	1
Bird	2	2	2
Crustacean	1	1	2
Fish	2	2	5
Insect	7	7	18
Reptile	1	1	1
**Mating success**	31	28	58
Collembola	1	1	1
Fish	1	1	2
Insect	29	26	55

### 2.3 Data analysis

All statistical analyses were performed in R version 3.6.1 (R Core Team, 2019), and figures were generated using the ‘ggplot2’ package (Wickham, 2016). Meta‐analyses were performed using Fisher's *z* transformation of the correlation coefficient (*Zr*). We then converted mean effect size estimates derived from our statistical models back to *r* for presentation in figures. Assumptions of linear mixed models were met for all models reported below.

#### 2.3.1 Main effects models

Using the function ‘rma.mv’ from the r package ‘metafor’ (Viechtbauer, 2010), we run a multilevel intercept‐only meta‐analytic model for each of our three traits of interest (mating latency, choosiness and mating success) to test for an overall effect of temperature. We ran both phylogenetic and non‐phylogenetic models to examine whether the evolutionary relationships between species influenced this overall effect. For the phylogenetic models, we first used the r package ‘rotl’ (Michonneau et al., 2016; OpenTree et al., 2021) to generate phylogenetic trees of the species included in our meta‐analysis (Figure S2). This tree was then imported into the ‘ape’ package (Paradis et al., 2004) and a correlation matrix obtained using the ‘vcv’ function. The resulting correlation matrix was then included in our multivariate meta‐analytic models as a random factor. We included additional random effects in our phylogenetic and non‐phylogenetic models to account for non‐independence due to the extraction of multiple effect sizes from the same study (study ID) and the use of the same species across studies (species ID). We also included a unit level random effect (effect size ID) as a measure of residual heterogeneity (Santos et al., 2011).

#### 2.3.2 Moderator models

Because phylogeny failed to resolve any of the heterogeneity, we did not include it in our moderator models (Santos et al., 2011; see Table S2). Moderators were tested using non‐phylogenetic multilevel meta‐regression models with study ID, species ID and effect size ID as random effects (Santos et al., 2011). For each of these models, we calculated *R* ^2^_marginal_, which describes the percentage of heterogeneity that was explained by the inclusion of a moderator (i.e. the estimated percentage decrease in heterogeneity between the main effects model and moderator model).

We first tested the effects of four moderators relating to the type of temperature treatment animals were exposed to in each study. We included a continuous moderator (‘intensity of temperature treatment’) to test whether variation in effect sizes could be explained by the extent of the temperature differences (°C) between treatments within each study. A categorical moderator (‘time of temperature treatment’) tested for differences between studies that exposed animals in early development, in adulthood before the mating trial, or during the mating trial. Another categorical moderator (‘type of temperature treatment’) tested for differences between studies that exposed animals to a short‐term versus a long‐term temperature treatment (i.e. acute exposure versus acclimation). We defined long‐term temperature treatment as any exposure to a different temperature that lasted more than 24 hr. For mating success, we included an additional categorical moderator (‘sex exposed to temperature treatment’) to test for differences between studies that exposed males, females, or both sexes to the temperature treatment.

We also tested the effects of two moderators relating to study methodology: (a) the choice paradigm used in each study (sequential or simultaneous mate choice trials) and (b) mating history (virgin or mated individuals). Lastly, we tested the effects of two moderators relating to species physiology or ecology: (a) habitat type (terrestrial or aquatic) and (b) whether the study used extreme temperatures outside the species' natural temperature range (yes or no). The latter was determined for each study based on the authors' own assessment of whether they were using extreme temperatures.

#### 2.3.3 Relationship between *Zr*_mating latency_ and *Zr*_mating success_

We also examined the relationship between effect sizes (*Zr*) for mating latency and mating success from studies that measured both traits. This was the case for 14 effect sizes from 10 studies on 9 different species. We analysed this reduced dataset using (a) a Pearson's correlation test between *Zr*_mating latency_ and *Zr*_mating success_, as well as (b) a meta‐regression with *Zr*_mating success_ as the response variable, *Zr*_mating latency_ as the moderator, and study ID, species ID and effect size ID as random effects.

#### 2.3.4 Publication bias tests

To examine the potential for underreporting of non‐significant results, we used the function ‘regrest’ to test for funnel plot asymmetry in our meta‐regression models (Nakagawa et al., 2017). We also tested for time‐lag bias using (a) a rank correlation test between effect size and publication year for each study and (b) a meta‐regression with publication year as a continuous moderator (Jennions & Møller, 2002).

### 2.4 Ethical note

This study did not require approval from an animal ethics committee, as it is a meta‐analysis of previously published studies.

## 3 RESULTS

We present mean effect size estimates derived from the statistical models with 95% confidence intervals in square brackets.

### 3.1 Publication bias tests

There was evidence for funnel asymmetry for mating success (*z* = −2.48, *p* = 0.013) but not for mating latency (*z* = 1.21, *p* = 0.23) or choosiness (*z* = 1.01, *p* = 0.31). This suggests a potential for publication bias in the mating success dataset (Figure S3). In addition, our models with publication year as a continuous moderator showed a time‐lag bias in the mating latency dataset (*Zr* = 0.03 [0.003, 0.05], *p* = 0.026) and the choosiness dataset (*Zr* = −0.04 [−0.06, −0.01], *p* = 0.004) but not the mating success dataset (*Zr* = −0.001 [−0.02, 0.02], *p* = 0.86). Similarly, the rank correlation test indicated significant variation in effect size over time for mating latency (*ρ* = 0.53, *p* = 0.003) and choosiness (*ρ* = −0.59, *p* < 0.001), but no overall trend for mating success (*ρ* = 0.03, *p* = 0.81). The trend for mating latency is from large negative effect sizes to an average effect size close to zero, whereas for choosiness, the trend is from small positive effect sizes to small negative effect sizes on average (Figure S4).

### 3.2 Main effects models

**FIGURE 1:**
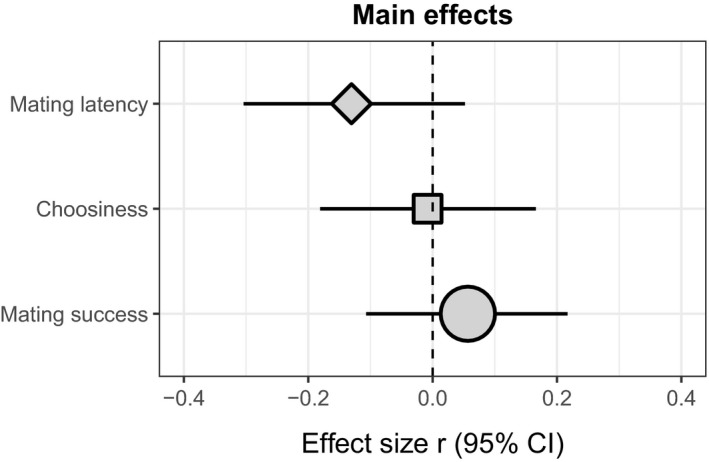
Mean effect size estimates derived from multilevel intercept‐only meta‐analytic models examining the effects of temperature on mating latency (diamond), choosiness (square) and mating success (circle). The relative size of each symbol represents the number of effect sizes included in that data set (mating latency = 29, choosiness = 29, mating success = 58).

Our intercept‐only models for mating latency (*Zr* = −0.13 [−0.31, 0.05]), choosiness (*Zr* = −0.01 [−0.18, 0.17]) and mating success (*Zr* = 0.06 [−0.11, 0.22]) showed no overall effect of temperature (Figure 1; Figures S5 and S6). For the mating latency model, the total heterogeneity (*I* ^2^) among the random factors was 92.5% with all of the variance attributed to between‐study differences. The rest of the random effects accounted for less than <0.1% of the total heterogeneity. For the choosiness model, the total heterogeneity was 84.6% with most of the variance attributed to between‐study differences (40.5%) and species differences (40.5%). Effect size identity and phylogeny accounted for 3.5% and <0.1% of the total heterogeneity, respectively. For the mating success model, the total heterogeneity was 94.2% with most of the variance attributed to between‐study differences (81.5%). Effect size identity accounted for the remaining 12.6% of the total heterogeneity, with less than 0.1% for phylogeny and between‐species differences. Since phylogeny failed to resolve any heterogeneity in our main effects models, the estimates from phylogenetic and non‐phylogenetic models were identical.

### 3.3 Moderator models

As in the main effects models, the estimates from the phylogenetic and non‐phylogenetic moderator models were similar or identical in all analyses. Statistical results reported below are based on the non‐phylogenetic models (see Table S2 for phylogenetic models).

**FIGURE 2:**
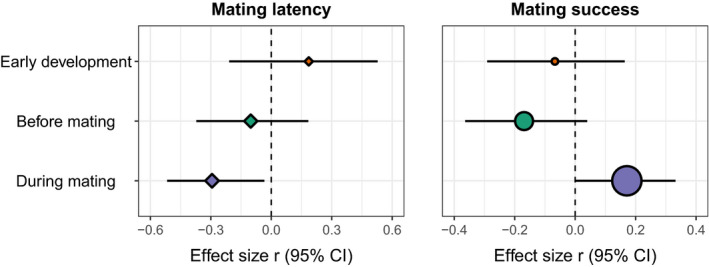
Mean effect size estimates derived from multilevel meta‐regression models examining how the ‘time of temperature treatment’ moderator (early development, adulthood before mating trial, or adulthood during mating trial) influences the relationship between temperature and mating latency (diamond) or mating success (circle). The relative size of each symbol represents the number of effect sizes included in that dataset (mating latency: early development = 6, before mating = 11, during mating = 12; mating success: early development = 6, before mating = 14, during mating = 38).

The time of temperature treatment did not have a statistically significant effect on mating latency (*Q*_*m*_ = 4.05, *p* = 0.13, *R* ^2^_marginal_ = 0.075; Figure 2). Mating latency was similar for animals exposed to a temperature treatment during early development (*Zr* = 0.19 [−0.21, 0.59]), in adulthood before mating trials (*Zr* = −0.10 [−0.39, 0.19]), or during mating trials (*Zr* = −0.30 [−0.57, −0.03]). The effects of temperature on mating latency also did not vary in response to the type of temperature treatment (acute exposure: *Zr* = −0.17 [−0.40, −0.06]; acclimation: *Zr* = −0.06 [−0.41, 0.28]; *Q*_*m*_ = 2.23, *p* = 0.33) or the intensity of the treatment (*Zr* = −0.01 [−0.04, 0.01], *Q*_*m*_ = 1.50, *p* = 0.22).

The relationship between temperature and choosiness was not influenced by the time of the temperature treatment (early development: *Zr* = 0.05 [−0.15, 0.26]; during mating trial: *Zr* = −0.04 [−0.23, 0.14]; *Q*_*m*_ = 1.32, *p* = 0.25). Similarly, this relationship did not vary based on the type of temperature treatment (acute exposure: *Zr* = −0.05 [−0.26, 0.15]; acclimation: *Zr* = 0.02 [−0.17, 0.21; *Q*_*m*_ = 0.84, *p* = 0.66) or the intensity of the temperature treatment (*Zr* = −0.03 [−0.06, 0.01]; *Q*_*m*_ = 2.59, *p* = 0.11).

The effect of temperature on mating success varied depending on whether the animals were exposed to a temperature treatment during early development (*Zr* = −0.07 [−0.30, 0.17]), in adulthood before mating trials (*Zr* = −0.17 [−0.38, 0.04]), or during mating trials (*Zr* = 0.17 [0.01, 0.34]). Higher temperatures during the mating trial were associated with a higher mating success (*Q*_*m*_ = 15.6, *p* < 0.001; Figure 2). This moderator (‘time of temperature treatment’) explained about 5% of the heterogeneity among effect sizes (*R* ^2^_marginal_ = 0.048). In contrast, the effects of temperature on mating success did not vary in response to the type of temperature treatment (acute exposure: *Zr* = 0.09 [−0.11, 0.29]; acclimation: *Zr* = −0.09 [−0.29, 0.11]; *Q*_*m*_ = 0.89, *p* = 0.64) or the intensity of the treatment (*Zr* = 0.01 [−0.02, 0.03], *Q*_*m*_ = 0.36, *p* = 0.55). Lastly, there were no substantial differences in effect sizes between studies that exposed males (*Zr* = −0.21 [−0.59, 0.17), females (*Zr* = −0.19 [−0.70, 0.33]), or both sexes (*Zr* = 0.09 [−0.10, 0.27]) to the temperature treatment (*Q*_*m*_ = 3.14, *p* = 0.37).

Lastly, there was no significant effect of the two moderators relating to study methodology (‘choice paradigm’ and ‘mating history’) on mating latency, choosiness, or mating success effect sizes (Table S4). Similarly, there was no significant effect of the two moderators relating to species physiology or ecology (‘habitat type’ and ‘extreme temperature’) on any of the three traits of interest (Table S4).

### 3.4 Relationship between *Zr*_mating latency_ and *Zr*_mating success_

**FIGURE 3:**
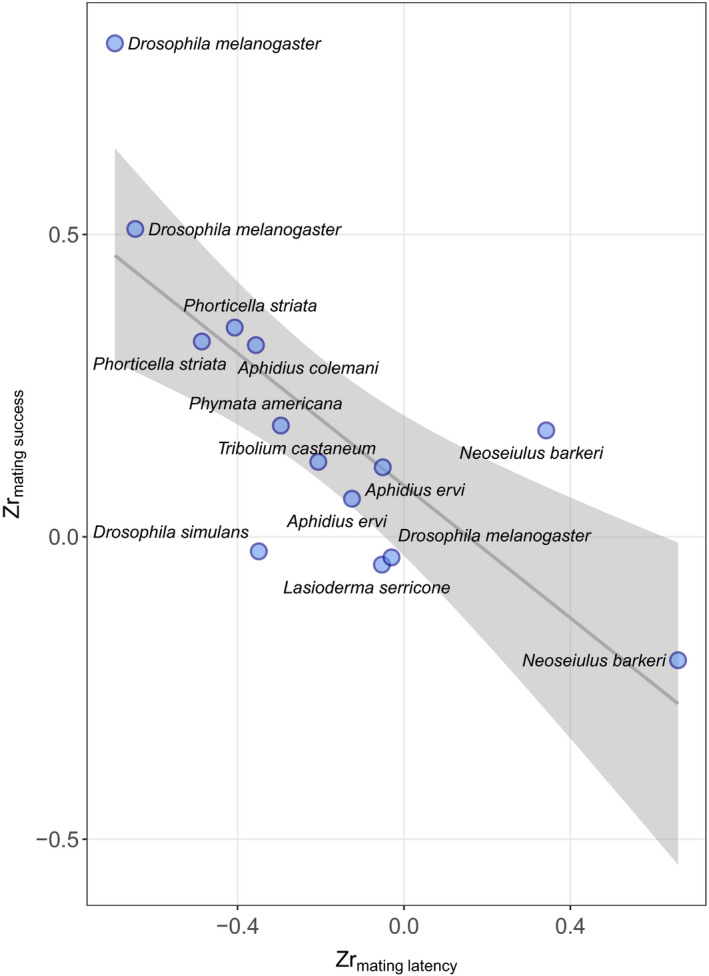
Fitted line and individual data points showing the relationship between *Zr*_mating latency_ and *Zr*_mating success_ for studies that measured both traits (14 effect sizes from 10 studies on 9 different species). The shaded area around the line of best fit indicates the 95% confidence interval.

We found a strong negative correlation between the effect sizes (*Zr*) for mating latency and mating success from studies that measured both traits (*r* = −0.77, *t*_12_ = −4.13, *p* = 0.001; Figure 3). Similarly, our meta‐regression showed that *Zr*_mating latency_ explained 75% of the heterogeneity in *Zr*_mating success_ (*Q*_*m*_ = 18.6, *p* < 0.0001, *R* ^2^_marginal_ = 0.745). This negative relationship indicates that in studies where a higher temperature led to an increase in mating latency, this was associated with a decrease in mating success, and vice versa (Figure 3).

## 4 DISCUSSION

Our meta‐analysis of 53 studies found no evidence for an overall effect of temperature on mating latency, choosiness, or mating success. There was an increase in mating success when animals were exposed to higher temperatures during mating trials, but not when they were exposed prior to that. Interestingly, in a subset of studies that measured both mating latency and mating success, we found a strong negative relationship between the effect sizes for these traits. This suggests that a decrease in mating latency at higher temperatures was associated with an increase in mating success. Overall, our findings suggest that temperature might affect the frequency of matings but not which mates they are choosing.

Such temperature‐induced changes in mating success may be mediated through physiological changes (Abram et al., 2017). The body temperature and metabolic rate of ectothermic animals are directly influenced by changes in ambient temperature, which can affect their activity levels (Gunderson & Leal, 2015; Kearney et al., 2010). This also means that higher temperatures might lead to increased costs of mate searching and mate assessment (García‐Roa et al., 2020; Punzalan et al., 2008). For example, ambush bugs (*Phymata americana*) have reduced mate‐searching success at higher ambient temperatures (Punzalan et al., 2008), which might result in a higher propensity to mate with a potential partner under these conditions.

Previous work has suggested that temperature may also affect mating success through changes in communication between mates (Martín & López, 2013). Mating latency and mating success are both dependent of the ability of a male to stimulate the female for mating. When mate communication involves chemical signals over large areas, such as femoral secretions to mark territories, females may take longer to detect them due to faster evaporation at higher temperatures (Martín & López, 2013). Such a disruption in mate communication may lead to a higher mating latency and lower mating success in warmer environments. On the other hand, when mate communication involves chemical signals at a small spatial scale, a higher temperature may increase the volatility of these chemicals and improve mate recognition and assessment. This may lead to a shorter mating latency and higher mating success in warmer environments. In line with this latter scenario, our meta‐analysis found evidence for an increase in mating success when animals were exposed to higher temperatures during mating trials, but it is not possible to determine whether the underlying mechanism for this pattern was a change in mate communication through chemical signals.

Contrary to our expectation, we found no evidence that temperature‐induced changes in mating success were accompanied with changes in mating latency or choosiness. This was somewhat surprising, given that mating latency, choosiness and mating success are closely linked (Breedveld & Fitze, 2015; Lindström & Lehtonen, 2013). For example, a choosier individual may require more time for mate assessment, leading to an increase in mating latency, which can then result in lower mating success (Hegde & Krishna, 1997). Similarly, a choosier individual may be less likely to mate with low‐quality partners, resulting in lower mating success. The absence of a directional effect of temperature on mating latency and choosiness, despite its effects on mating success, may be partly due to the use of different datasets for each of these traits (Table 1). In fact, only one of the studies in our meta‐analysis included data on all three traits (Table S2). In a subset of studies that measured both mating latency and mating success, there was a strong negative relationship between the effect sizes for these traits, as we had expected (Figure 3). This supports our interpretation that the different patterns we observed in mating latency, choosiness and mating success may be due to differences in the species used across the three datasets. A recent review argued that the relationship between temperature and sexual selection is likely to vary across species in relation to their mating system, physiology and behaviour (García‐Roa et al., 2020). The absence of overall directional effects in mating behaviour in our meta‐analysis provides support for this prediction. Importantly, our findings suggest that due to substantial among‐species variation, it may be difficult to generate predictions for how the strength of sexual selection in natural populations will change in a warming world.

Phylogeny did not seem to influence the effects of temperature on mating behaviour across the range of species included in our analysis (mating latency: *n* = 14, choosiness: *n* = 14, choosiness: *n* = 28). This may be because certain features of our datasets make the detection of a phylogenetic signal unlikely. For example, mating behaviour has the capacity to evolve rapidly and is thus evolutionarily labile (Blomberg et al., 2003; Dougherty & Shuker, 2015). Our analysis also includes measures of mating preference (choosiness) for a wide range of traits, such as body size, colouration and mating calls, which might make it more difficult to find a phylogenetic signal. Lastly, it is worth noting that a large majority of studies included in our analysis were on insects (Table 1), limiting our ability to draw conclusions about general patterns across taxa. For example, we initially intended for our meta‐analysis to include a comparison between endotherms and ectotherms. We expected that temperature changes would have a stronger effect on mating behaviour and mating success in ectotherms, given that ambient temperature can directly affect their body temperature, metabolic rate, locomotor performance and activity levels (Gibert et al., 2001; Gunderson & Leal, 2015; Kearney et al., 2010; Lachenicht et al., 2010). However, it was not possible to carry out this comparison due to the relevant studies available in published literature. Out of 53 studies included in our meta‐analysis, only two were on endotherms (birds). We therefore strongly encourage future research on the effects of temperature on mating behaviour and mating success in endotherms, as well as ectotherms other than insects.

Our publication bias tests suggest there may be some influence on the overall results. Firstly, there was evidence for funnel asymmetry for mating success, suggesting a potential for publication bias in this dataset. The observed bias may have also been caused by unexplained heterogeneity among studies due to other moderators that we did not consider in our analysis. Secondly, we found evidence for a time‐lag bias in the mating latency and choosiness datasets, where there was a trend for a decrease in effect size over time (Figure S4). This is a common pattern in meta‐analyses in ecology and evolutionary biology (Jennions & Møller, 2002). We have not used methods such as trim and fill that try to compensate for publication bias, as they may perform poorly in high heterogeneity datasets due to poor coverage probability and potentially misleading adjustments (Moran et al., 2020; Moreno et al., 2009). Regardless of the underlying causes for this publication bias, it is important to take it into consideration when interpreting the results, particularly in cases where confidence intervals are close to zero.

Another limitation of our study was that the sample sizes for the mating latency (*k* = 29) and choosiness datasets (*k* = 29) were relatively small. As a result, subset analyses testing for the effects of moderators had small sample sizes for each factor (Table S1). Most of these analyses did not detect any significant effects, with the exception of an effect of the time of temperature treatment on mating success, which was discussed above. The fact that the intensity and duration of the temperature treatment did not have an overall effect on mating behaviour and mating success was surprising and may be due to low statistical power.

Our meta‐analysis focused on the effects of temperature on mating success and two precopulatory traits, mating latency and choosiness. Nevertheless, temperature can also influence postcopulatory processes (García‐Roa et al., 2020). For example, the amount and quality of sperm transferred during mating has been shown to vary with temperature (Gasparini et al., 2018; Reinhardt et al., 2015; Sales et al., 2018; Walsh et al., 2019). A study on the cigarette beetle *Lasioderma serricone* that examined both precopulatory and postcopulatory traits actually found that temperature had a stronger effect on the latter (Suzaki et al., 2018). We therefore suggest that a meta‐analysis on how temperature variation also influences postcopulatory traits, such as sperm production and sperm competition, may be worthwhile.

Given rising temperatures due to global climate change, it is important to better understand how changes in temperature may affect mating patterns and sexual selection (García‐Roa et al., 2020). Here, we show an increase in mating success when animals were exposed to higher temperatures during mating trials, but not during early development or in adulthood before the mating trials. We found no evidence for directional effects of temperature on mating latency or choosiness, suggesting it may be difficult to generate general predictions for how the strength of sexual selection will change in a warming world. Nevertheless, we also found a strong negative relationship between the effect sizes for mating latency and mating success. This suggests that in species where a higher temperature leads to an increase in mating latency, this may result in a decrease in mating success, and vice versa. Our meta‐analysis therefore provides new insights into the effects of temperature on mating behaviour and sexual selection.

## AUTHORS' CONTRIBUTIONS

N.P. conceived the study; A.B. and N.P. carried out the data extraction and data analysis; N.P. led the writing of the manuscript and A.B. provided comments on the manuscript draft. Both authors gave final approval for publication.

## CONFLICT OF INTEREST

The authors have no conflict of interest.

## Supporting information


Appendix S1

## Data Availability

All data used in this meta‐analysis and the associated R code are available from the Dryad Digital Repository 10.5061/dryad.jwstqjqc8 (Pilakouta & Baillet, 2022).
