# Supplementary material for: Effects of temperature on mating behaviour and mating success: A meta‐analysis
Source: J Anim Ecol. 2022 Jul 10;91(8):1642–50. doi: 10.1111/1365-2656.13761 (PMC9541322; doi:10.1111/1365-2656.13761)
Supplement: Supplementary file 1 — Appendix S1 [file JANE-91-1642-s001.pdf]

Supplementary Table 1. Number of effect sizes (*k*) in our data sets on mating latency, choosiness, and mating success.

	Mating latency	Choosiness	Mating success
Sex			
Female	19	22	42
Male	0	5	2
Both	10	2	14
Time of temperature treatment			
Early development	6	13	6
Before mating	11	0	14
During mating	12	16	38
Type of temperature treatment			
Acute exposure	20	9	29
Acclimation	9	20	29

Supplementary Table 2. Summary of data extracted from each study used in the meta-analysis. For each study, we present an abbreviated reference to the study, the scientific name of the study species, and the types of data extracted from that study (i.e., mating latency, choosiness, and/or mating success).

**Study**	**Species**	**Mating latency**	**Choosiness**	**Mating success**
Albrecht *et al.* (1999)	*Pomacea canaliculate*	x		
Amin *et al.* (2010)	*Bombus terrestris*			x
Arbogast (2007)	*Plodia interpunctella*			x
Beaulieu & Sockman (2012)	*Melospiza lincolnii*		x	
Beckers & Schul (2008)	*Neoconocephalus triops*		x	
Brandt *et al.* (2018)	*Habronattus clypeatus*			x
Caetano & Hajek (2017)	*Sirex noctilio*			x
Colinet & Hance (2009)	*Aphidius colemani*	x		x
Conrad *et al.* (2017)	*Osmia bicornis*			x
Coomes *et al.* (2019)	*Taeniopygia guttata*		x	
Delisle (1995)	*Choristoneura rosaceana*			x
Dubey *et al.* (2016)	*Menochilus sexmaculatus*		x	
Everman *et al.* (2018)	*Drosophila melanogaster*	x		x
Fasolo & Krebs (2004)	*Drosophila melanogaster, Drosophila simulans, Drosophila mojavensis*			x
Geister & Fischer (2007)	*Bicyclus anynana*			x
Gerhardt (2005)	*Hyla chrysoscelis*		x	
Goebel (2006)	*Chilo sacchariphagus*			x
Grace & Shaw (2004)	*Laupala cerasina*		x	
Hsu & Wu (2001)	*Ctenocephalides felis*			x
Ingleby *et al.* (2013)	*Drosophila simulans*	x		x
Ismail *et al.* (2010)	*Aphidius ervi*	x	x	x
Janowitz & Fischer (2011)	*Bicyclus anynana*	x		
Jiao *et al.* (2009)	*Pardosa astrigera*	x		
Kindle *et al.* (2006)	*Gryllodes sigillatus, Acheta domestricus*			x
Kvanermo & Forsgren (2000)	*Pomatoschistus minutus*		x	
Laudien & Seifert (1983)	*Drosophila simulans*			x
McKibben & Bass (1998)	*Porichthys notatus*		x	

Mhatre *et al.* (2011)	*Oecanthus henryi*	x		
Milner *et al.* (2010)	*Uca mjoebergi*		x	
Olvido *et al.* (2010)	*Allonemobius socius*		x	
Papadopoulou (2006)	*Lasioderma serricone*			x
Parkash *et al.* (2011)	*Drosophila melanogaster*	x		
Patton & Krebs (2001)	*Drosophila melanogaster, Drosophila simulans, Drosophila mojavensis*			x
Pires & Hoy (1992)	*Gryllus firmus*	x		
Punzalan *et al.* (2008)	*Phymata americana*	x		x
Putz & Crews (2005)	*Eublepharis maculatus*		x	
Ritchie *et al.* (2001)	*Drosophila montana*		x	
Sambucetti & Norry (2015)	*Drosophila buzzatii*			x
Scharf *et al.* (2019)	*Tribolium castaneum*	x		x
Sih *et al.* (2002)	*Aquarius remiges*			x
Singh *et al.* (2016)	*Drosophila melanogaster*	x		x
Stazione *et al.* (2019)	*Drosophila melanogaster*	x		
Suzaki *et al.* (2018)	*Lasioderma serricone*	x		x
Symes *et al.* (2017)	*Oecanthus forbesi*		x	
Vasudeva *et al.* (2018)	*Callosobruchus maculatus*			x
Westerman & Monteiro (2016)	*Bicyclus anynana*	x		
Wilson *et al.* (2007)	*Gambusia holbrooki*			x
Yang *et al.* (2017)	*Nilaparvata lugens*			x
Yenisetti *et al.* (2006)	*Phorticella striata*	x		x
Zhang *et al.* (2013)	*Plutella xylostella*	x		x
Zhang *et al.* (2016)	*Neoseiulus barkeri*	x		
Zizzari & Ellers (2011)	*Orchesella cincta*			x
Zverev *et al.* (2018)	*Chrysomela lapponica*			x

Supplementary Table 3. Results of phylogenetic meta-regression models on mating latency, choosiness, and mating success. The moderators tested were time of temperature treatment (early development, in adulthood before mating, or during mating), type of temperature treatment (acute exposure or acclimation), and the intensity of the treatment. Statistically significant values are indicated in bold. Phylogeny failed to resolve any of the heterogeneity in our models, so the results from the phylogenetic and non-phylogenetic moderator models were similar or identical in all analyses.

	Mating latency	Choosiness	Mating success
Time of temperature treatment	Q_m=4.05<br>P=0.13	Q_m=1.32<br>P=0.25	Q_m=15.6<br>**P<0.001**
Type of temperature treatment	Q_m=2.23<br>P=0.33	Q_m=0.85<br>P=0.66	Q_m=0.89<br>P=0.64
Intensity of temperature treatment	Q_m=1.50<br>P=0.22	Q_m=2.59<br>P=0.11	Q_m=0.36<br>P=0.55

Supplementary Table 4. Results of meta-regression models assessing the effects of four moderators on the effect sizes of mating latency, choosiness, and mating success. For each model, we report the levels of the moderators along with number of effect sizes (*k*) for each level. Omnibus Q test of moderators (Q_m) and the p-value (*p*) were obtained from models with the intercept. Point estimates (*Zr*) and 95% confidence intervals (CI) were obtained from models without intercepts. Omnibus Q tests were not possible for moderators where there was information only for one level (e.g., mating history: virgins vs NA). In those cases, we provide point estimates and 95% CI for models without NA values. We also could not test the effect of temperature extremes in the choosiness dataset, because all of those studies were done within the species' natural temperature range.

**Moderator**	**Moderator levels (*k*)**	Q_m	*p*	*Zr*	**95% CI**
**Mating latency**					
Mating history	Virgins (*k*=26)<br>NA (*k*=3)	—	—	–0.09	[–0.27, 0.10]
Habitat type	Terrestrial (*k*=27)<br>Aquatic (*k*=2)	2.89	0.09	–0.10<br>–0.79	[–0.28, 0.08]<br>[–1.57, -0.01]
Extreme temperature	No (*k*=19)<br>Yes (*k*=10)	0.01	0.94	–0.13<br>–0.13	[–0.34, 0.07]<br>[–0.36, 0.11]
**Choosiness**					
Choice paradigm	Simultaneous mate choice (*k*=20)<br>Sequential mate choice (*k*=5)<br>NA (*k*=4)	0.37	0.54	–0.04<br>0.10	[–0.28, 0.21]<br>[–0.27, 0.47]
Mating history	Virgins (*k*=13)<br>Mated (*k*=2)<br>NA (*k*=14)	1.71	0.19	–0.003<br>0.47	[–0.33, 0.32]<br>[–0.16, 1.09]
Habitat type	Terrestrial (*k*=24)<br>Aquatic (*k*=5)	0.03	0.87	–0.01<br>0.04	[–0.21, 0.18]<br>[–0.52, 0.60]
**Mating success**					
Mating history	Virgins (*k*=45)<br>NA (*k*=13)	—	—	0.04	[–0.16, 0.24]
Habitat type	Terrestrial (*k*=56)<br>Aquatic (*k*=2)	0.01	0.91	0.06<br>0.01	[–0.11, 0.23]<br>[–0.91, 0.92]
Extreme temperature	No (*k*=48)<br>Yes (*k*=10)	0.42	0.52	0.08<br>–0.05	[–0.10, 0.27]<br>[–0.42, 0.32]

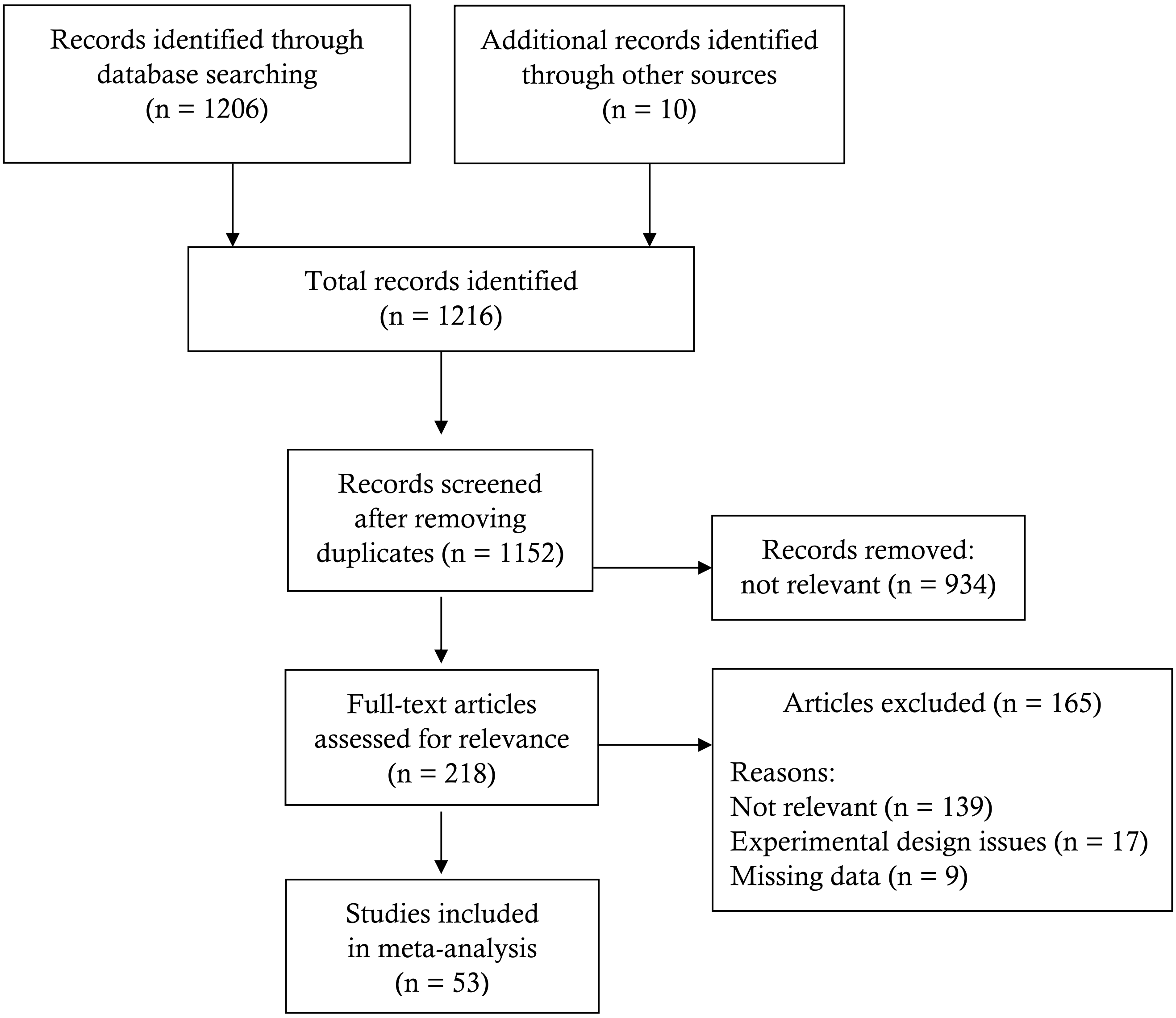

Supplementary Figure 1. PRISMA diagram showing the selection process for the studies included in this meta-analysis.

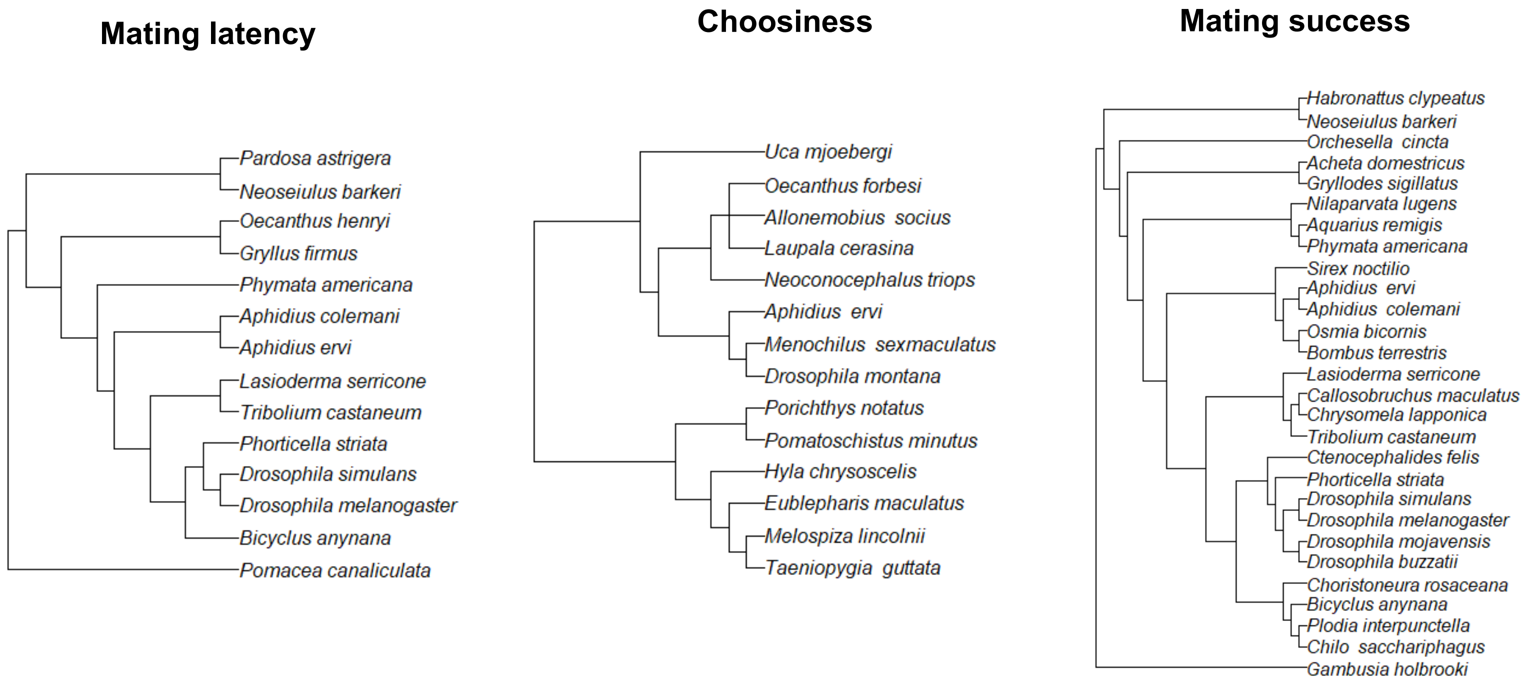

Supplementary Figure 2. Phylogenetic trees for the species included in the mating latency, choosiness, and mating success datasets used in the meta-analysis.

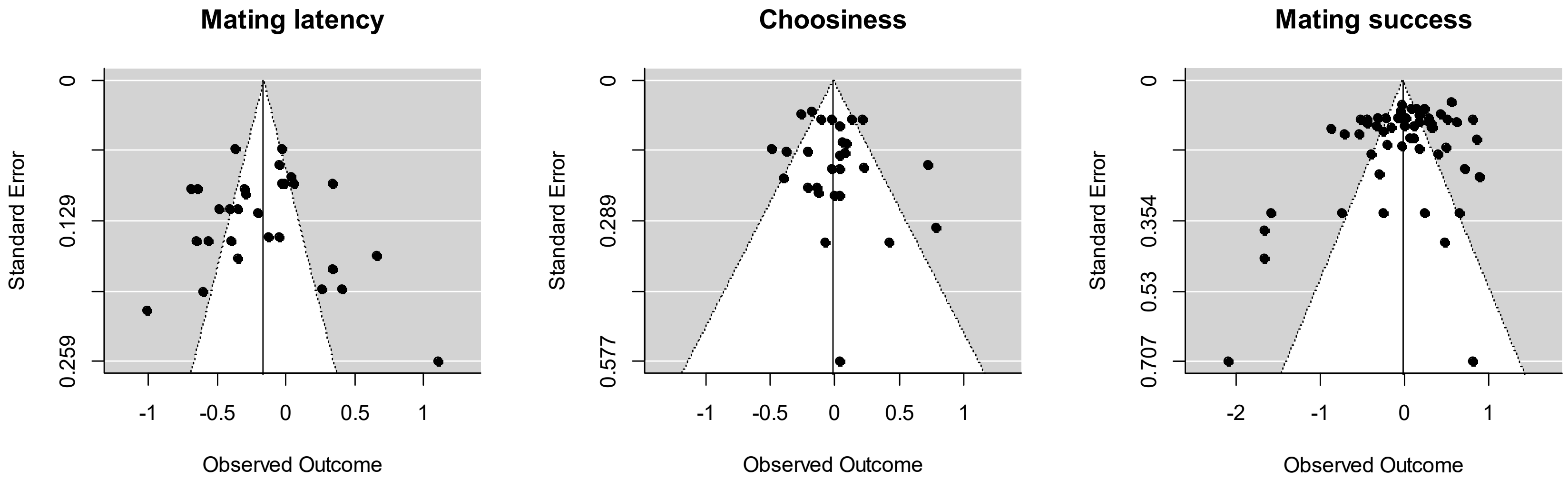

Supplementary Figure 3. Funnel plots generated to examine the potential for underreporting of non-significant results in each of our three datasets (mating latency, choosiness, and mating success).

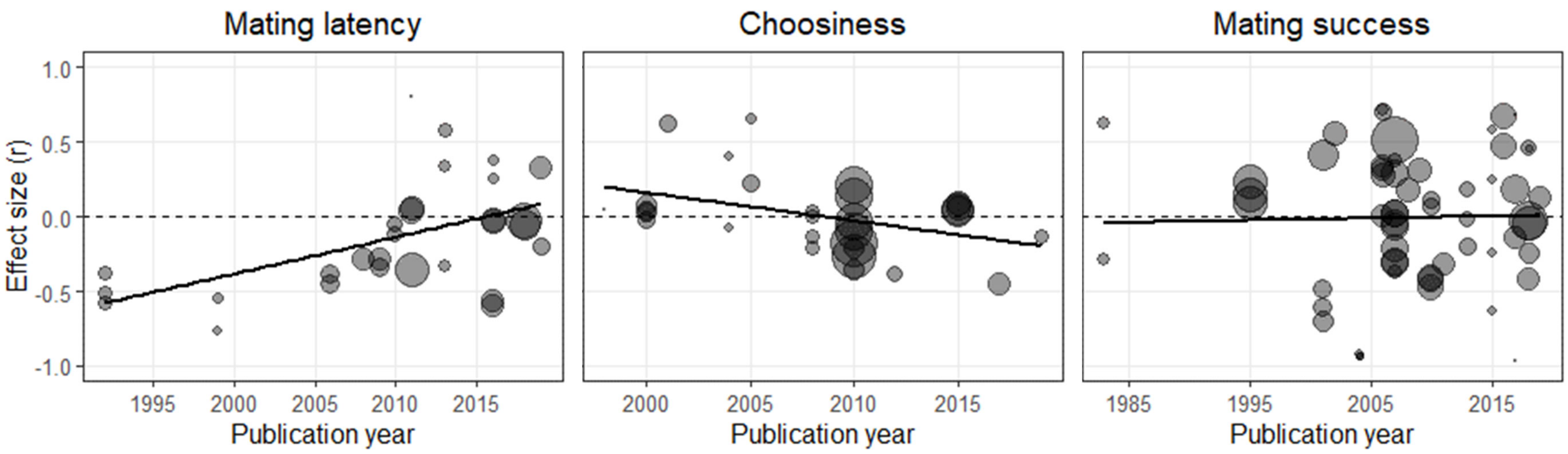

Supplementary Figure 4. Effect size (r) of the relationship between temperature and mating latency, choosiness, or mating success over time. The relative size of each point represents the sample size of each effect size. The solid black line represents the regression of effect size by publication year.

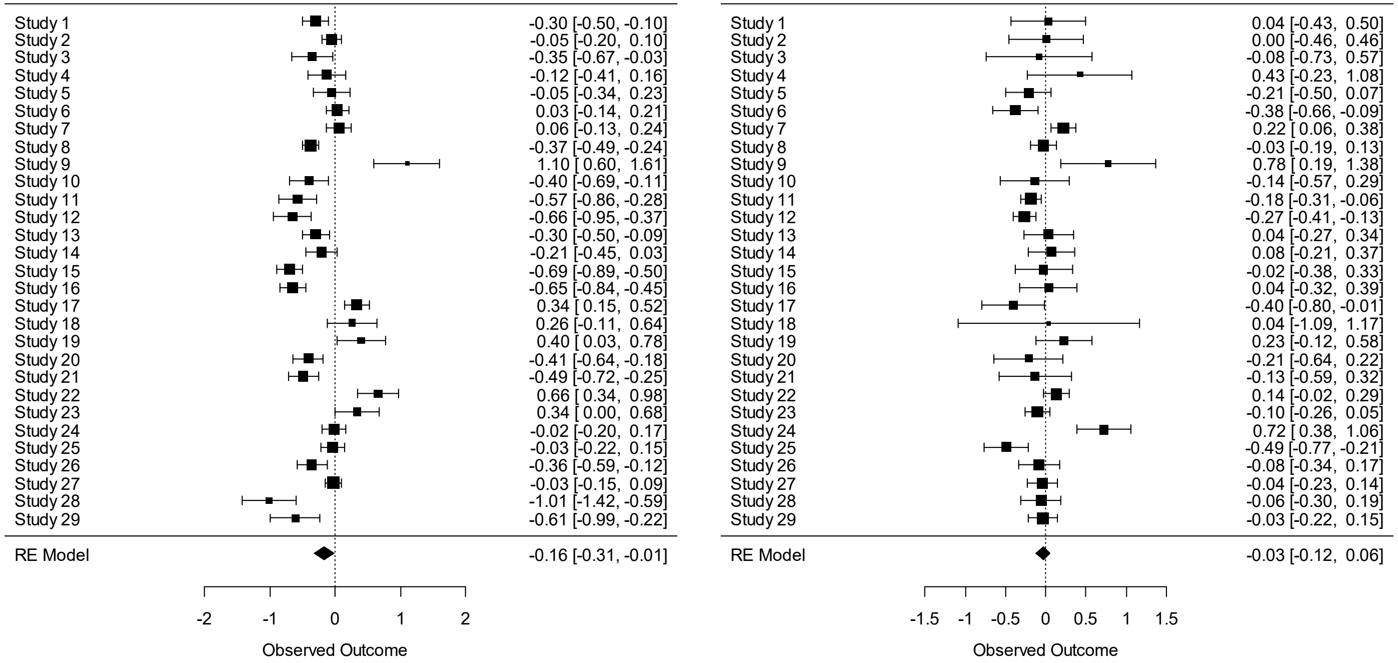

Supplementary Figure 5. Forest plots for mating latency (left) and choosiness (right).

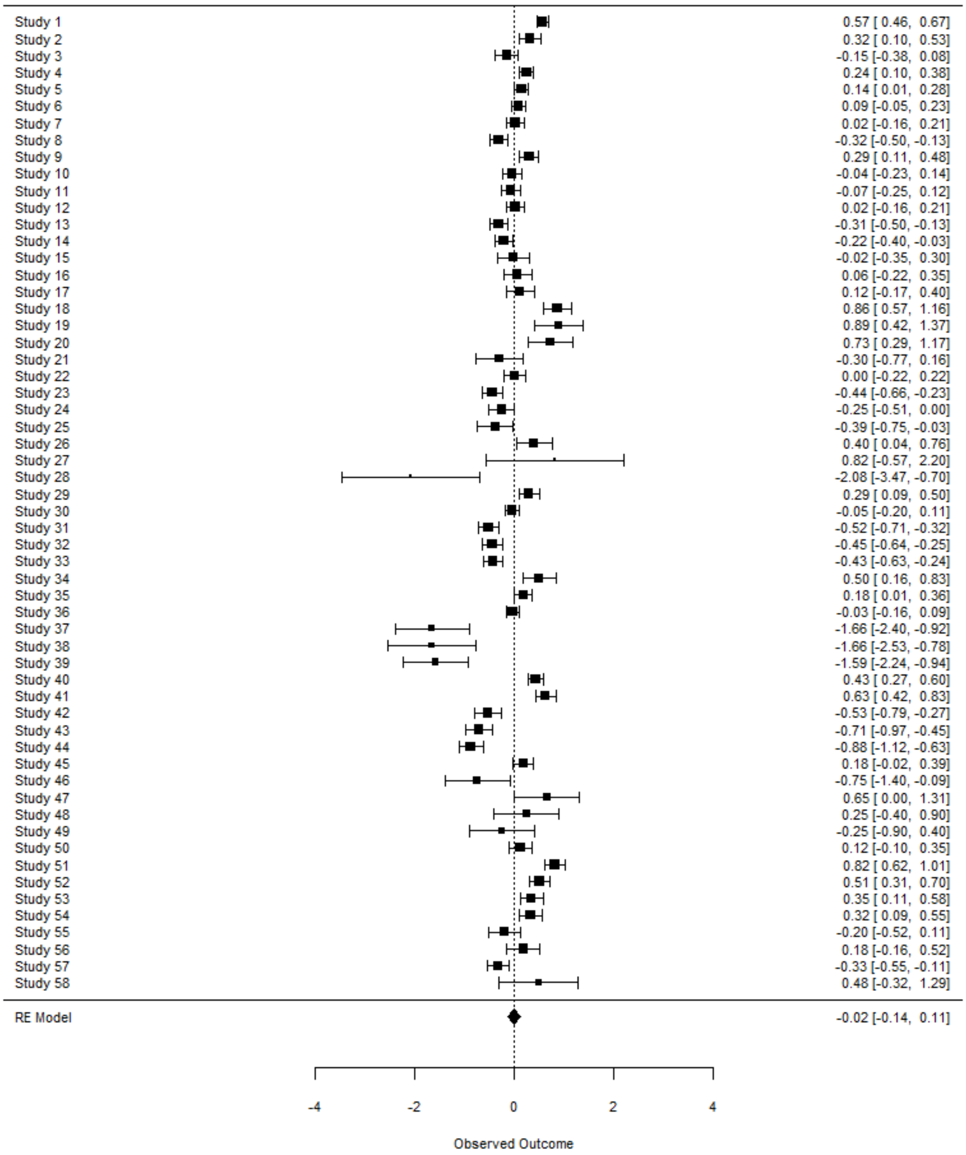

Supplementary Figure 6. Forest plot for mating success.

Supplementary References

Albrecht, E. A., Carreño, N. B., & Castro-Vazquez, A. (1999). A quantitative study of environmental factors influencing the seasonal onset of reproductive behaviour in the South American apple-snail *Pomacea canaliculata* (Gastropoda: Ampullariidae). *Journal of Molluscan Studies*, 65(2), 241–250. https://doi.org/10.1093/mollus/65.2.241

Amin, M. R., Than, K. K., & Kwon, Y. J. (2010). Mating status of bumblebees*, Bombus terrestris* (Hymenoptera: Apidae) with notes on ambient temperature, age and virginity. *Applied Entomology and Zoology*, 45(3), 363–367.https://doi.org/10.1303/aez.2010.363

Arbogast, R. T. (2007). A wild strain of *Plodia interpunctella* (Hübner) (Lepidoptera: Pyralidae) from farm-stored maize in South Carolina: effect of temperature on mating, survival, and fecundity. *Journal of Stored Products Research*, 43(4), 503–507.

Beaulieu, M., & Sockman, K. W. (2012). Song in the cold is "hot": memory of and preference for sexual signals perceived under thermal challenge. *Biology Letters*, 8(5), 751–753. https://doi.org/10.1098/rsbl.2012.0481

Beckers, O. M., & Schul, J. (2008). Developmental plasticity of mating calls enables acoustic communication in diverse environments. *Proceedings of the Royal Society B: Biological Sciences*, 275(1640), 1243–1248. https://doi.org/10.1098/rspb.2007.1765

Brandt, E. E., Kelley, J. P., & Elias, D. O. (2018). Temperature alters multimodal signaling and mating success in an ectotherm. *Behavioral Ecology and Sociobiology*, 72(12). https://doi.org/10.1007/s00265-018-2620-5

Caetano, I. A. L., & Hajek, A. E. (2017). Mating behavior and sexual receptivity of *Sirex noctilio* (Hymenoptera: Siricidae). *Annals of the Entomological Society of America*, saw095. https://doi.org/10.1093/aesa/saw095

Colinet, H., & Hance, T. (2009). Male reproductive potential of *Aphidius colemani* (Hymenoptera: Aphidiinae) exposed to constant or fluctuating thermal regimens. *Environmental Entomology*, 38(1), 242–249. https://doi.org/10.1603/022.038.0130

Conrad, T., Stöcker, C., & Ayasse, M. (2017). The effect of temperature on male mating signals and female choice in the red mason bee, *Osmia bicornis* (L.). *Ecology and Evolution*, 7(21), 8966–8975. https://doi.org/10.1002/ece3.3331

Coomes, C. M., Danner, R. M., & Derryberry, E. P. (2019). Elevated temperatures reduce discrimination between conspecific and heterospecific sexual signals. *Animal Behaviour*, 147, 9–15. https://doi.org/10.1016/j.anbehav.2018.10.024

Delisle, J. (1995). Effect of male and female age on the mating success of the obliquebanded leafroller *Choristoneura rosaceana* (Lepidoptera: Tortricidae) under different ecological conditions. *Journal of Insect Behavior*, 8(6), 781–799.

Dubey, A., Omkar, & Mishra, G. (2016). Influence of temperature on reproductive biology and phenotype of a ladybird, *Menochilus sexmaculatu*s (Fabricius) (Coleoptera: Coccinellidae). *Journal of Thermal Biology*, 58, 35–42.

Everman, E. R., Delzeit, J. L., Hunter, F. K., Gleason, J. M., & Morgan, T. J. (2018). Costs of cold acclimation on survival and reproductive behavior in *Drosophila melanogaster. Plos One*, 13(5), e0197822. https://doi.org/10.1371/journal.pone.0197822

Fasalo, A. G., & Krebs, R. A. (2004). A comparison of behavioural change in *Drosophila* during exposure to thermal stress. *Biological Journal of the Linnean Society*, 83(2), 197–205. https://doi.org/10.1111/j.1095-8312.2004.00380.x

Geister, T. L., & Fischer, K. (2007). Testing the beneficial acclimation hypothesis: temperature effects on mating success in a butterfly. *Behavioral Ecology*, 18(4), 658–664. https://doi.org/10.1093/beheco/arm024

Gerhardt, H. C. (2005). Advertisement-call preferences in diploid-tetraploid treefrogs (*Hyla chrysoscelis* and *Hyla versicolor*): implications for mate choice and the evolution of communication systems. *Evolution, 59(2), 395–408.* https://doi.org/10.1111/j.0014-3820.2005.tb00998.x

Goebel, R. (2006). The effect of temperature on development and reproduction of the sugarcane stalk borer, *Chilo sacchariphagus* (Bojer 1856) (Lepidoptera: Crambidae). *African Entomology*, 14(1), 00–00.

Grace, J. L., & Shaw, K. L. (2004). Effetcs of developmental environment on signal-preference coupling in a Hawaiian cricket. *Evolution*, 58(7), 1627–1633.

Hsu, M. H., & Wu, W. J. (2001). Off-host observations of mating and postmating behaviors in the cat flea (Siphonaptera: Pulicidae). *Journal of Medical Entomology*, 38(3), 352–360. https://doi.org/10.1603/0022-2585-38.3.352

Ingleby, F. C., Hunt, J., & Hosken, D. J. (2013). Genotype-by-environment interactions for female mate choice of male cuticular hydrocarbons in *Drosophila simulans*. *Plos One*, 8(6), e67623. https://doi.org/10.1371/journal.pone.0067623

Ismail, M., Vernon, P., Hance, T., & van Baaren, J. (2010). Physiological costs of cold exposure on the parasitoid *Aphidius ervi*, without selection pressure and under constant or fluctuating temperatures. *BioControl*, 55(6), 729–740.

Janowitz, S. A., & Fischer, K. (2011). Opposing effects of heat stress on male versus female reproductive success in *Bicyclus anynana* butterflies. *Journal of Thermal Biology*, 36(5), 283–287. https://doi.org/10.1016/j.jtherbio.2011.04.001

Jiao, X., Wu, J., Chen, Z., Chen, J., & Liu, F. (2009). Effects of temperature on courtship and copulatory behaviours of a wolf spider *Pardosa astrigera* (Araneae: Lycosidae). *Journal of Thermal Biology*, 34(7), 348–352.

Kindle, T. K., Johnson, K. M., Ivy, T. M., Weddle, C. B., & Sakaluk, S. K. (2006). Female mating frequency increases with temperature in two cricket species, *Gryllodes sigillatus* and *Acheta domesticus* (Orthoptera: Gryllidae). *Canadian Journal of Zoology*, 84(9), 1345–1350. https://doi.org/10.1139/z06-127

Kvarnemo, C., & Forsgren, E. (2000). The influence of potential reproductive rate and variation in mate quality on male and female choosiness in the sand goby, *Pomatoschistus minutus. Behavioral Ecology and Sociobiology*, 48(5), 378–384. https://doi.org/10.1007/s002650000246

Laudien, H., & Seifert, U. (1983). Influence of breeding- and experimental-temperature on courtship and copulation in *Drosophila simulans. Journal of Thermal Biology*, 8(4), 435–437. https://doi.org/10.1016/0306-4565(83)90044-X

McKibben, J. R., & Bass, A. H. (1998). Behavioral assessment of acoustic parameters relevant to signal recognition and preference in a vocal fish. *The Journal of the Acoustical Society of America*, 104(6), 3520–3533. https://doi.org/10.1121/1.423938

Mhatre, N., Bhattacharya, M., Robert, D., & Balakrishnan, R. (2011). Matching sender and receiver: poikilothermy and frequency tuning in a tree cricket. *Journal of Experimental Biology*, 214(15), 2569–2578. https://doi.org/10.1242/jeb.057612

Milner, R. N. C., Detto, T., Jennions, M. D., & Backwell, P. R. Y. (2010). Experimental evidence for a seasonal shift in the strength of a female mating preference. *Behavioral Ecology*, 21(2), 311–316. https://doi.org/10.1093/beheco/arp196

Olvido, A. E., Fernandes, P. R., & Mousseau, T. A. (2010). Relative effects of juvenile and adult environmental factors on mate attraction and recognition in the cricket, *Allonemobius socius. Journal of Insect Science*, 10(90), 1–17.

Papadopoulou, S. (2006). Observations on the mating behavior of *Lasioderma serricorne* (F.) adults and experiments on their nutritional requirements in dried tobacco. *The Coleopterists Bulletin*, 60(4), 291–296.

Parkash, R., Sharma, V., Chahal, J., Lambhod, C., & Kajla, B. (2011). Impact of body melanization on mating success in *Drosophila melanogaster. Entomologia Experimentalis et Applicata*, 139(1), 47–59. https://doi.org/10.1111/j.1570-7458.2011.01102.x

Patton, Z. J., & Krebs, R. A. (2001). The effect of thermal stress on the mating behavior of three *Drosophila* species. *Physiological and Biochemical Zoology*, 74(6), 783–788. https://doi.org/10.1086/323327

Pires, A., & Hoy, R. R. (1992). Temperature coupling in cricket acoustic communication. *Journal of Comparative Physiology A*, 171(1), 69–78.

Punzalan, D., Rodd, F. H., & Rowe, L. (2008). Sexual selection mediated by the thermoregulatory effects of male colour pattern in the ambush bug *Phymata americana. Proceedings of the Royal Society B: Biological Sciences*, 275(1634), 483–492.

Putz, O., & Crews, D. (2005). Embryonic origin of mate choice in a lizard with temperature-dependent sex determination. *Developmental Psychobiology*, 48(1), 29–38.

Ritchie, M. G., Saarikettu, M., Livingstone, S., & Hoikkala, A. (2001). Characterization of female preference functions for *Drosophila montana* courtship song and a test of the temperature coupling hypothesis. *Evolution*, 55(4), 721–727.

Sambucetti, P., & Norry, F. M. (2015). Mating success at high temperature in highland- and lowland-derived populations as well as in heat knock-down selected *Drosophila buzzatii. Entomologia Experimentalis et Applicata*, 154(3), 206–212.

Scharf, I., Wertheimer, K.-O., Xin, J. L., Gilad, T., Goldenberg, I., & Subach, A. (2019). Context-dependent effects of cold stress on behavioral, physiological, and life-history traits of the red flour beetle. *Insect Science, 26, 142–153.*

Sih, A., Lauer, M., & Krupa, J. J. (2002). Path analysis and the relative importance of male–female conflict, female choice and male–male competition in water striders. *Animal Behaviour*, 63(6), 1079–1089. https://doi.org/10.1006/anbe.2002.2002

Singh, K., Samant, M. A., Tom, M. T., & Prasad, N. G. (2016). Evolution of pre- and post-copulatory traits in male *Drosophila melanogaster* as a correlated response to selection for resistance to cold stress. *Plos One*, 11(4), e0153629. https://doi.org/10.1371/journal.pone.0153629

Stazione, L., Norry, F. M., & Sambucetti, P. (2019). Heat-hardening effects on mating success at high temperature in *Drosophila melanogaster. Journal of Thermal Biology*, 80, 172–177. https://doi.org/10.1016/j.jtherbio.2019.02.001

Suzaki, Y., Kodera, S., Fujiwara, H., Sasaki, R., Okada, K., & Katsuki, M. (2018). Temperature variations affect postcopulatory but not precopulatory sexual selection in the cigarette beetle. *Animal Behaviour*, 144, 115–123.

Symes, L. B., Rodríguez, R. L., & Höbel, G. (2017). Beyond temperature coupling: effects of temperature on ectotherm signaling and mate choice and the implications for communication in multispecies assemblages. *Ecology and Evolution*, 7(15), 5992–6002. https://doi.org/10. 1002/ece3.3059

Vasudeva, R., Deeming, D. C., & Eady, P. E. (2018). Larval developmental temperature and ambient temperature affect copulation duration in a seed beetle. *Behaviour*, 155(1), 69–82. https://doi.org/10.1163/1568539X-00003479

Westerman, E., & Monteiro, A. (2016). Rearing temperature influences adult response to changes in mating status. *Plos One*, 11(2), e0146546.

Wilson, R. S., Hammill, E., & Johnston, I. A. (2007). Competition moderates the benefits of thermal acclimation to reproductive performance in male eastern mosquitofish. *Proceedings of the Royal Society B: Biological Sciences*, 274(1614), 1199–1204. https://doi.org/10.1098/rspb. 2006.0401

Yang, B.-J., Liu, M.-L., Zhang, Y.-X., & Liu, Z.-W. (2017). Effects of temperature on fitness costs in chlorpyrifos-resistant brown planthopper, *Nilaparvata lugens* (Hemiptera: Delphacidae). *Insect Science*, 25(3), 409–417. https://doi.org/10.1111/1744-7917.12432

Yenisetti, S. C., Hegde, S. N., Venkateswarlu, M., & Krishna, M. S. (2006). Phenotypic plasticity of sexual behavior at different temperatures in a Drosophilid: *Phorticella striata*. *Korean J. Genetics*, 28(4), 395-401.

Zhang, G. H., Li, Y. Y., Zhang, K. J., Wang, J. J., Liu, Y. Q., & Liu, H. (2016). Effects of heat stress on copulation, fecunditiy and longevity in newly-emerged adults of the predatory mite *Neoseiulus barkeri* (Acari: Phytoseiidae). *Systematic and Applied Acarology*, 21(3), 295. https://doi.org/10.11158/saa.21.3.5

Zhang, W., Zhao, F., Hoffmann, A. A., & Ma, C.-S. (2013). A single hot event that does not affect survival but decreases reproduction in the diamondback moth, *Plutella xylostella. Plos One*, 8(10), e75923. https://doi.org/10.1371/journal.pone.0075923

Zizzari, Z. V., & Ellers, J. (2011). Effects of exposure to short-term heat stress on male reproductive fitness in a soil arthropod. *Journal of Insect Physiology*, 57(3), 421–426. https://doi.org/10.1016/j.jinsphys.2011.01.002

Zverev, V., Kozlov, M. V., Forsman, A., & Zvereva, E. L. (2018). Ambient temperatures differently influence colour morphs of the leaf beetle *Chrysomela lapponica*: Roles of thermal melanism and developmental plasticity. *Journal of Thermal Biology*, 74, 100–109. https://doi.org/10.1016/j.jtherbio.2018.03.019
